# Are We Publishing “The Right Stuff”?

**DOI:** 10.1371/journal.pmed.0030512

**Published:** 2006-11-28

**Authors:** The PLoS Medicine Editors, The PLoS Medicine Editors

## Abstract

The findings of a major study in*PLoS Medicine* predicting mortality rates and the global burden of disease should inform health policy, and also the future content of medical journals.

This month we publish the findings of a major study estimating the likely trends in global morbidity and mortality [1]. Such a study should surely cause us, as editors, to pause and reflect on the implications for what we should be publishing. Which medical conditions will be the major causes of death and disease, and is *PLoS Medicine* publishing key research studies addressing those conditions? Ideally, as a general medical journal, the proportion of research on different diseases that we publish should be roughly equivalent to their contribution to the global burden of disease. In other words, we have to ask, “Are we publishing the right stuff?”

Mathers and Loncar [1] have updated the much-cited Global Burden of Disease (GBD) study published a decade ago [2]. They have produced alternative baseline, optimistic, and pessimistic projections for mortality and for the burden of disease, measured in terms of disability-adjusted life years (DALYs). Researchers, policy makers, and others will often need to consult these revised projections and we are delighted that the authors have chosen to publish them in an open-access journal. An important feature of many *PLoS* articles is that supplementary files are also freely available. The supporting documents accompanying the Mathers and Loncar article contain a wealth of detailed information and it is essential that such data is placed in the public domain.

With minor modifications, Mathers and Loncar have adopted the same methodology as was used in the earlier GBD study. This is not a radical new “take” on the issue and we should not be surprised that no major changes in the GBD estimates have emerged.

However, the findings confirm that the burden of morbidity and mortality that is due to HIV/AIDS will be substantially higher than estimated in the original GBD study. Small declines in the total burden of morbidity and mortality due to a number of other causes will result in the overall projections for mortality for 2030 remaining much the same as predicted earlier. In the baseline projection, by 2015, HIV/AIDS will be the leading cause of burden of disease in both low- and middle-income countries, but the predicted rise in tobacco-attributable deaths means that, globally, tobacco will kill 50% more people than HIV/AIDS. The three leading causes of burden of disease in 2030 are projected to be HIV/AIDS, unipolar depressive disorders, and ischemic heart disease. Road traffic accidents are the fourth leading cause in the baseline scenario, and the third leading cause, ahead of ischemic heart disease, in the optimistic scenario.

An inspection of the research published in *PLoS Medicine* in recent months shows that we are indeed giving due attention to HIV/AIDS and to other infectious diseases. But we need to publish more research on cardiovascular disease, diabetes, mental health, road traffic accidents, and chronic obstructive pulmonary disease.

It has been interesting to observe that some medical specialties more than others have chosen to submit their work to us. In particular, researchers from the infectious diseases, epidemiology, and public health communities have shown strong support for the journal. One reason for this support might be that, for example, AIDS researchers—all too aware that HIV is most prevalent in the world's poorest countries—have more swiftly grasped the need to make the results of research freely available to all. Nonetheless, the advantages of open access apply to all disciplines.

But the implications of the findings of Mathers and Loncar obviously go considerably beyond the content of this or any other journal. Their report should help set the agenda for policy and establish the priorities for research. But will it? Sadly, it is all too clear that the greatest needs are generally not those that receive the greatest attention. Most notably, only 10% of expenditure on health research and development is devoted to the problems that primarily affect the poorest 90% of the world's population [3].

Mathers and Loncar caution that continued economic and social development is required for further progress to be made in reducing the global burden, and they stress that they have worked on the assumption that such development will lead to improved health in the developing world, in the same manner that it has done in richer nations. Global climate change and the dwindling of key resources (notably oil) could very well hold back economic growth and put development efforts off-track. There are many “unknowns”—including international conflict—that can never be factored into the calculations of those who are brave enough to seek to predict the future.

Things could be much worse than the most pessimistic of the report's three alternative projections, most notably if the levels of economic growth that they have assumed are not attained. But they could also be much better than even the most optimistic projection. This would require many institutions and individuals also to “do the right stuff.” Identifying the problems is a good place to start, but it must now lead on to the development of constructive policies and to collaborative action.

## Abbreviations

GBD: Global Burden of Disease
